# Efficacy of engineered GO Amberlite XAD-16 picolylamine sorbent for the trace determination of Pb (II) and Cu (II) in fishes by solid phase extraction column coupled with inductively coupled plasma optical emission spectrometry

**DOI:** 10.1038/s41598-018-35656-1

**Published:** 2018-12-03

**Authors:** Hina Javed, Aminul Islam, Anjali Chauhan, Suneel Kumar, Sushil Kumar

**Affiliations:** 1 Analytical Research Laboratory, Department of Chemistry, Aligarh Muslim University, Aligarh, 202002 India; 2 School of Environmental Sciences, Jawaharlal Nehru University, New Delhi, 110067 India

## Abstract

Graphene oxide (GO) was immobilized innovatively through azo spacer arm onto the surface of polymeric Amberlite XAD-16 resin in order to expose all oxygen functionalities freely available for metal ions coordination and further modification with picolylamine which governs selectivity. The GO Amberlite XAD-16 picolylamine enables the development of SPE column coupled with ICP-OES for preconcentration and determination of Pb (II) and Cu (II) in water and fish samples. Elution was performed by mild acid (2M HCl) no other carcinogenic organic solvent was used, prevents ligand leaching. Under optimized conditions, the preconcentration factors of 150 and detection limits 1.434 and 0.048 µg L^−1^ for Pb (II) and Cu (II)  were obtained respectively.

## Introduction

Graphene Oxide (GO), one of the derivatives of graphene^1–3^, but now has written its own history in the field of material chemistry such as environmental applications of water treatment^4,5^. GO readily disperses in water to form stable colloidal dispersions, facilitating mainly its carboxyl, hydroxyl groups to readily dissociate into their anionic forms in aqueous systems^6–9^. It maintains its negative surface charge down to very low values of pH. Combining with this huge surface area to mass ratio provided by the sheets, attributed to the fact that, why investigations into the use of GO as an adsorbent material has a plethora of the removal of toxic metals from aquatic environments^6,8^. In spite of many standard analytical methods for the determination of metal ions in real samples, development of another new selective method is at the forefront due to variation of sample matrices and concentration level of analyte which influences interferences and detection limit parameters respectively. Analytical techniques with superior detection limits frequently require separation methods to eliminate interferences and less sensitive one need preconcentration^10^. Solid phase extraction (SPE) addresses both these issues along with advantages such as low cost, high efficiency, rapid phase separation, ease of operation, excellent recovery of the sorbed analyte with small volumes of mineral acids rather than toxic organic solvents and reusability^11^. Moreover, this method can be easily incorporated with other spectroscopic techniques like FAAS, ICP-OES etc. in offline and online modes. ICP-OES exhibits higher sensitivity, large dynamic linear range, low detection limits, and simultaneous analysis^12^. Despite the aforementioned facts, determination of the trace metals in environmental samples by ICP-OES constitutes one of the major problems of interferences in direct determination. Therefore, their determination is coupled with SPE in order to exclude all interfering matrices. GO serve as a potential candidate over graphene or any other currently reported sorbents like CNT^13–16^, sulfur nanoparticle^17^, activated carbon^18^ etc. due to its ultra-high sorption capacities for various metal ions^19^. This is attributed to the fact that GO has an exceptional morphology and properties, all carbon atoms are characterized by oxygen functionalities on its either side in a planar geometry, renders its hydrophilicity, chemical reactivity and huge surface area^20,21^. These groups coordinate with metal ions through sharing of a lone pair of electrons, exposing it’s both sides for coordination to the available surrounding and enabling fast metal ion sorption and elution^22^. Although, huge success has been achieved by employing GO as an ideal sorbent, yet possesses some limitations. For instance, separation and recycling of GO for its direct and multiple uses, becomes challenging. GO tends to aggregate in a batch method, due to the strong van der Waals and π-π stacking which can significantly affect the efficiency and reusability of GO as it requires sonication and ultracentrifugation before its use in the next sorption cycle and also the complete collection of GO sheets from aqueous solution is insurmountable even with the 0.22 µm cellulose membrane^23^. Henceforth, fabrication with magnetic nanoparticles Fe_3_O_4_^24^ and further modification through covalent bonding with TETA^25^, Polyimide^26^ has somehow been able to resolve these issues of using direct GO in batch technique. Magnetic nanoparticles occupy some surface sites on GO and reduces binding sites available for metal ions resulting in a decrease in sorption capacity^27^ and number of regeneration cycles^28,29^. Moreover, the restriction of using large sample volume for metal ion preconcentration remains a challenge in batch method. These problems can be resolved by choosing a column method owing to its significant number of theoretical plates needed for good extraction, higher preconcentration factor, large number of regeneration cycles and possible on-line automation with different determination techniques^30^. However, SPE column loaded with GO sheets generates high back pressure and may escape to the environment and has been found to report Parkinson’s disease like symptoms in zebra fish larvae^31^. Efforts were made to deal with these limitations by immobilizing GO through covalent coupling onto a solid support such as silica^22^ while glycidylmethacrylate^32^ and polystyrene divinylbenzene via spacer arm by utilising its carboxylic functionalities^23^. One of the drawbacks of utilizing carboxylic group through solid support is that its oxygen group will not be available for coordination to metal ions as well as for further functionalization through a ligand in order to get selectivity. Therefore, a novel and innovative approach is developed by immobilizing GO onto the surface of Amberlite XAD-16 resin through azo coupling with the objective of exposing all freely available oxygen functionalities for metal ion coordination and further modification with picolylamine in order to improve selectivity. The synthesized sorbent was employed for the development of SPE column coupled with ICP-OES for preconcentration and determination of Pb (II) and Cu (II) in both water sample as well as different animal tissues (muscle, liver, brain, kidney etc.) of fishes. Fishes are preferred because of their longer survival in polluted water bodies and have more chances of metal contamination.

## Results and Discussion

### Characterization

**Figure 1:**
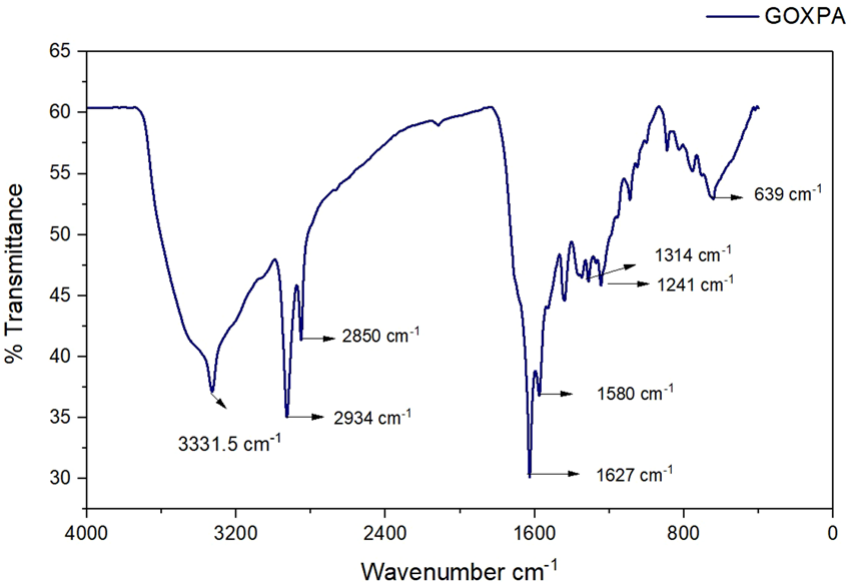
FT-IR spectrum of GOXPA.

**Figure 2:**
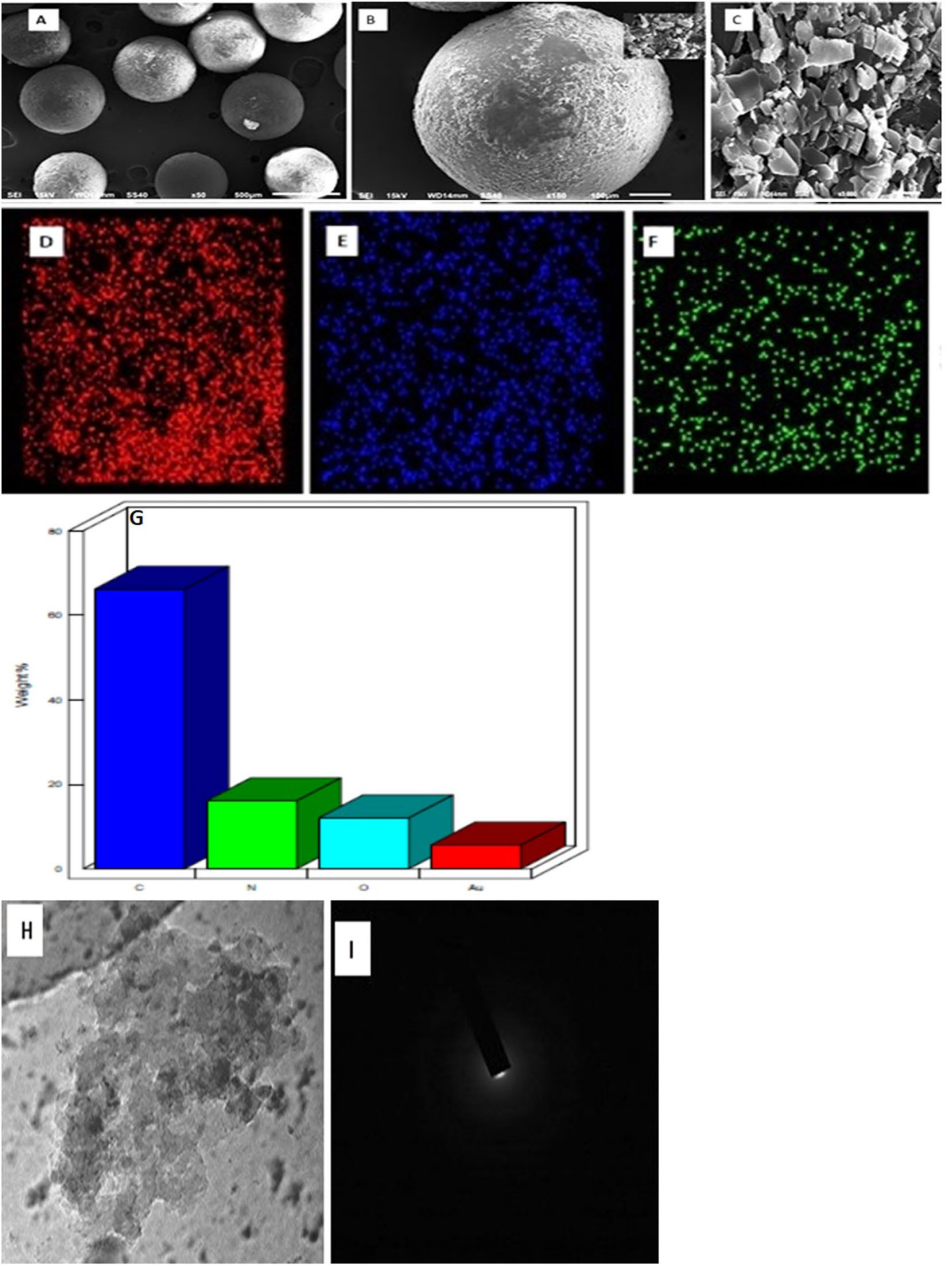
SEM images of (**A**) cluster of GOXPA resin beads (**B**) single GOXPA resin bead (**C**) GO sheets (**D**–**G**) Elemental mapping images of C, O and N respectively (**H**) TEM image of GOXPA (**I**) SAED pattern.

To study the surface modification of GO, the FT-IR spectra of (GO AXAD-16 picolylamine) GOXPA was inspected (Fig. 1). The most prominent sharp peak at 3331 cm^−1^ is attributed to NH stretching vibration of amide bond indicating the coupling of GO and picolylamine^33,34^. The band at 1580 cm^−1^ assigned to the azo (-N=N) stretching which confirmed the immobilization of GO onto AXAD-16 resin through azo spacer arm^33,34^. The peak at 1627 cm^−1^ corresponds to (-C=O). Additionally, peaks structured at 1314, 1241 and 759 cm^−1^, are associated with stretching of (-C-N, -C-O and pyridine ring) vibration of picolylamine respectively^35^. Moreover, the peak appearing at 639 cm^−1^ is assigned to (-N-H) wagging. The immobilization of GO onto the AXAD −16 resin was also confirmed by SEM micrograph. As it can be seen, the surface of AXAD-16 was disorderly covered by GO nano sheets (Fig. 2A–C). The EDS results (Fig. 2D–G) procured from SEM images of GOXPA and their elemental mapping showing spots of homogenous distribution of C (66.15%), N (16.12%), O (12.08%) revealing the presence of azo spacer arm and picolylamine. Furthermore, appearance of GO onto AXAD-16 was also confirmed by TEM image (Fig. 2H). In the selected area electron diffraction (SAED) pattern of GOXPA (Fig. 2I) the diffraction dots are not present, indicating the amorphous nature of GOXPA, which may describe the presence of oxygen functionalities on GO surface. The surface area of GOXPA investigated after adsorption and desorption of nitrogen using BET method, was found to be 134 m^2^ g^−1^ which was higher than reported earlier for the immobilized GO on to the solid support^23,32,36^. ^13^C NMR of GOXPA was also analyzed and observed a chemical shift in the spectrum showing the presence of amide bond (155 ppm), -CH_2_-NHR (45 ppm), benzylic −C−NHR (38 ppm), styrenic −CH and −CH_2_ (23,33 ppm), carboxylic carbon (165 ppm), -C-N=N-C- (126 ppm)^23,32^ (Supplementary Fig. S1). The TGA/DTA curve of GOXPA depicts a significant weight loss due to the desorption of adsorbed interstitial water molecules around 170 °C. Then GOXPA exhibit gradual decrease in weight up to 210 °C which may be attributed to the loss of CO and CO_2_ from decomposition of its oxygen functionalities which was supported by DTA endothermic curve suggesting its stability up to 210 °C. Then, a sharp weight loss observed around 420 °C illustrates lowering in its thermal stability (Supplementary Fig. S2).

### Optimization

#### pH Effect

It is experimentally found that the metal ion sorption is governed by protonation and deprotonation of available binding sites of functional groups of a sorbent and metal species present in the aqueous solution. Moreover, the suitable pH not only raises the adsorption capacity of metal ion but also reduces the interferences of coexisting ions. Therefore, pH plays a key role in the adsorption and desorption of metal ion. The efficacy of adsorption of Pb (II) and Cu (II) on to the GOXPA was revealed by plotting sorption capacity as a function of pH (Supplementary Fig. S3). The surface charge of GOXPA was further explained by point zero charge studied by the procedure as reported in the previous literature^32^. The point zero charge obtained from the plot of ∆pH vs pH_i_ was found to be 4.3. If pH < pH_pzc_ then surface of a sorbent is positively charged and if pH > pH_pzc_ then surface is negatively charged^32^. Thus, the surface of GOXPA is negatively charged and it shows electrostatic interactions with metal ions. At higher acidic condition the adsorption capacity decreases because the binding sites on GOXPA were protonated and undergo electrostatic repulsion when a metal species approached to it, resulting in poor adsorption of Pb (II) and Cu (II). However, the adsorption capacity increases from 3.6 to 4.6 and shows maximum adsorption at pH 5.6. This is attributed to the fact that the positively charged density generated on binding sites now starts decreasing and left partially unprotonated resulting in the decrease in repulsion between binding sites of GOXPA and the metal cation. Pb (II) and Cu (II) found in different forms at different pH values. Here the predominated species are (Pb^2+^ and PbOH^+^, and Cu^2+^, CuOH^+^). Following higher pH (>8) it was investigated that Pb shows higher sorption capacity which is due to the contribution of co precipitation (Pb (OH)_2_ of Pb (II) apart from electrostatic and chelate effect. Hence, pH 5.6 was used for further experimental studies of both Pb (II) and Cu (II)^37^. Although, the adsorption capacity of GO was reported to be very high for transition metals^19^. However, when GO is incorporated on to support, capacity was shown to be decreased^22,32,38^ since incorporation of a support material on to GO is requested in order to resolve some limitations attached with the direct use of GO in column.

#### Sample flow rate

**Figure 3:**
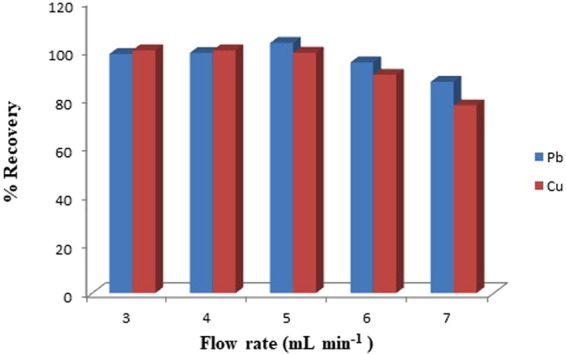
Sample (GOXPA) flow rate.

The optimization of a sample flow rate is an important parameter for the sorption and elution of Pb (II) and Cu (II) onto the GOXPA. Therefore, effect of flow rate of sample solution was investigated by passing 5 µg of Pb (II) and Cu (II) in 50 mL of an aqueous solution buffered at pH 5.6 through the column in the range of 3–7 mL min^−1^ (Fig. 3). In all flow rates Pb (II) observed to be kinetically faster than Cu (II). It was observed that both metal ions could be recovered quantitatively (>98%) in the range of flow rate 3–5 mL min^−1^. At the flow rate of 5 mL min^−1^ recoveries were found to be 103 and 99% for Pb (II) and Cu (II), respectively. However, at a higher flow rate of 7 mL min^−1^ efficiency was reduced to 87% and 77.3% this may be due to the less interaction time of Pb (II) and Cu (II) with the sorbent. To enhance the analytical speed, an eluent flow rate of 5.0 mL min^−1^ was optimized in this work.

### Elution studies and Reusability

**Figure 4:**
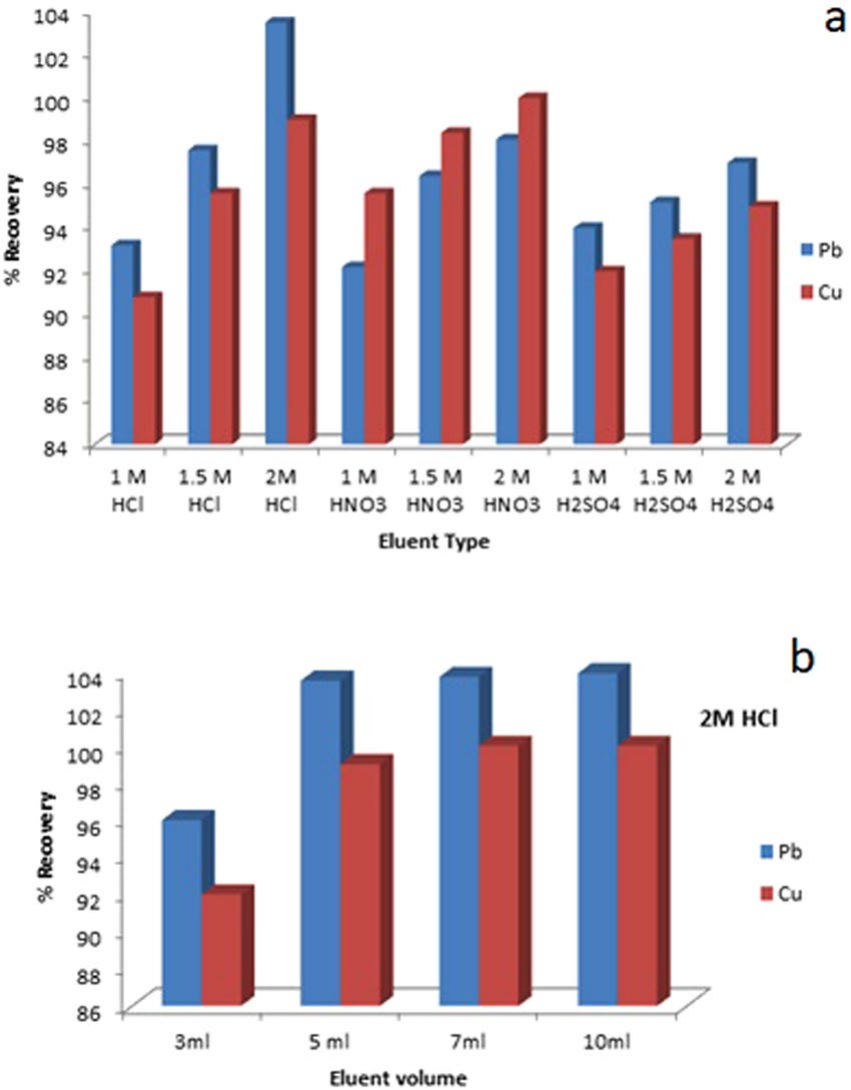
Elution studies (**a**,**b**).

To evaluate the effect of eluent type, concentration and volume on the recovery studies of Pb (II) and Cu (II), various eluting agents such as HCl, HNO_3_ and H2SO_4_ of different concentrations(1,1.5 and 2 M) were employed while keeping the elution volume constant i.e. 5 mL. Elution studies revealed that Pb (II) and Cu (II) can be quantitatively eluted by 5 mL of 2 M HCl with recovery of 103 and 99% respectively which is highest among the eluting agents tested. The interaction between an analyte and a chelating group can be easily destroyed by protonation of available binding sites leading to the elution of the analyte. Since HCl and HNO_3_ can easily dissociate their H^+^ ion in aqueous solution and can form an ionic bond with metal ion than H_2_SO_4_ acid. Such a low volume and concentration (5 mL of 2M HCl) gave efficient recoveries. Quantitative elution was not achieved by smaller volume (<5 mL) and lower concentration (<2 M). However, it was attained by 7 to 10 mL of 2M HCl but at the expense of preconcentration factor. In order to get a good preconcentration factor volume of an eluent is kept as small as possible. Therefore, 5 mL of 2M HCl was used for elution studies. As shown in graph (Fig. 4). Reusability test was performed on the GOXPA for the successive sorption and elution of analytes having concentration 100 µg L^−1^, volume 50 mL at a 5 mL min^−1^. The results indicated that the resin can be used for quantitative sorption and elution up to 50 cycles as no significant loss in sorption capacity was found and >96% recoveries were obtained. This may be attributed to the structural rigidity of the sorbent which is due to the presence of azo spacer arm. Additionally, the elution was performed by using mild acid (2M HCl) so it does not cause any leaching of ligand. However, slight loss in capacity was assessed for both Pb (II) and Cu (II) (94 and 91% recoveries) respectively for over 55 cycles. It was concluded that the resin GOXPA, could be re-used up to 50 cycles.

#### Interference studies

The real sample usually contains more than one metallic species. Therefore, it is necessary to evaluate the sorption behavior under competitive conditions, which may be either through a redox reaction or by competition for complex formation. In general, cationic matrices compete for the active binding sites of the sorbent with the analyte ion whereas anionic part will compete for analyte with sorbent resulting in a decrease of sorption capacity of target metal ion. The interference to analyte ratio for various alkali, alkaline earth and transition metal ions was investigated by preconcentrating a trace quantity of Pb (II) and Cu (II) (5 µg), 0.1 g resin, pH 5.6, flow rate 5 mL min^−1^ in the presence of a large amount of interfering ions. It was found that no significant effect was there on preconcentration of Pb (II) and Cu (II) up to (3–6 folds) of the added ions as the recovery was >96% for both ions (Table 1).

**Table 1:** Effect of Foreign species under optimized conditions.

Interfering ions	Added as	Interferent to analyte ratio	Mean % Recovery Cu (II)	RSD^a^ N = 3	Mean % Recovery Pb (II)	RSD N = 3
Cd	CdCl_2_	3	99.7	0.56	99.5	0.71
		6	96.0	1.47	98.0	1.44
Ni	Ni(NO_3_)_2_	3	99.0	1.42	100.2	0.28
		6	95.8	1.18	99.5	0.42
Co	Co(NO_3_)_2_	3	99.2	0.21	99.9	0.14
		6	96.1	3.09	98.6	0.57
Zn	ZnCl_2_	3	99.3	0.42	99.8	0.14
		6	98.5	0.71	99.3	0.14
Cu	Cu(NO_3_)_2_	3	—	—	98.7	0.14
		6	—	—	97.4	0.36
Pb	Pb(NO_3_)_2_	3	97.1	0.43	—	—
		6	97.0	1.45	—	—
NaCl	Na^+^	5 × 10^4^	102.4	0.82	103.7	0.13
	Cl^−^	2 × 10^4^	99.9	0.14	100.2	0.56
Na_2_SO_4_	SO4^2^^−^	1 × 10^4^	99.8	1.13	99.9	1.55
KCl	k^+^	1 × 10^4^	103.2	0.82	100.5	0.14
MgCl_2_	Mg^2+^	2 × 10^4^	103.3	0.13	101.2	1.39

^a^Relative standard deviation.

#### Preconcentration

Direct determination of metal ions in a real sample is insurmountable due to their low concentration therefore needs preconcentration prior to their determination. In order to study the quantitative recovery of Pb (II) and Cu (II) by keeping the metal ions loading constant at 5 µg, we continuously increase the sample volume and found the quantitative recoveries of Pb (II) and Cu (II) up to a sample volume of 750 mL (101.1 and 99.2%). When the volume was further increased to 1000 mL, the recoveries were reduced to (92.2 and 89.7%) for Pb (II) and Cu (II) respectively. Hence, the preconcentration factor was calculated as 150 with a preconcentration limit of 6.7 µg L^−1^ for both Pb (II) and Cu (II).

#### Comparative studies of GOXPA with other SPE method

Although, the adsorption capacity of GO was reported very high for transition metals^19^. However, when GO is incorporated on to solid support, capacity was shown to decreased^22,32,38^ since, incorporation of a support material on to GO is requested in order to resolve some limitations attached with direct use of GO in column or batch. This is quiet paramount that only a small quantity of GO is immobilized onto a long polymeric styrene support material through azo spacer arm without utilizing any oxygen functionality, used in column operation. The results of the current study based on some separation/preconcentration of Pb (II) and Cu (II) are compared with other proposed method (Table 2). The comparative data indicating the superiority of our work over others regarding sorption capacity, detection limit, preconcentration factor, elution studies of the system apart from inherent advantages of column operation and reusability.

**Table 2:** Comparison of figure of merits with previously reported GO based column SPE method.

Sorbents	Analytes	SC^a^ (mg g^−1^)	LOD^b^ (µg L^−1^)	Eluents	PF^c^	Techniques	Applications	Reference
GOXPA	Pb, Cu	51.8,17.4	1.43, 0.048	5 mL (2M HCl)	150	ICP-OES	Battery effluent, Fish samples	This work
Dithizone immobilized silica gel	Cu	0.76	0.2	10% HNO3	43	FAAS	Water samples	^42^
Sulfur nanopartice	Cu, Pb	3.55, 4.69	0.24, 0.63	3 mL(3.0M HNO3) in methanol	158.0, 175.0	FAAS	Marine samples	^43^
GO-TBCPA	Cu	6.0	0.63	3M HNO3	250	FAAS	Water, blood, tomato, spinach and soil samples	^44^
GO@SiO2	Pb, Cu	13.6,6.0	0.27,0.084	2 mL (2M HNO3)	200, 250	FAAS	water sample	^22^
Graphene with dithizone	Pb	16.6	0.61	2 mL (2M HNO3)	125	FAAS	water and vegetable samples	^45^
GO-TiO2	Cu, Pb,	0.8, 13.5,	0.48, 2.64	1.5 mL (0.5M HNO3)	10	ICP-OES	Environmental sample	^46^
GO-MCNTs-DETA	Pb	6.6	0.24	2 mL (2M HCl)	75	ICP-OES	Wastewater samples	^38^
Polycarboxylic microsphere polymer gel	Cu, Pb	—	0.01,0.02	2M HNO3	50–150	FAAS, ETAAS	Sea water, Mineral water	^47^
Nanometer-sized ZrO2	Cu	1.3	0.058	3M HCl	25	ICP-OES	Dried fish and water	^12^
Dithizone modified TiO2 nanoparticle	Pb	22.5	1.72	1.5 mL (0.25M HCl)	—	ICP-AES	Food stuff & Plant sample	^48^
Acrylic acid grafted polytetrafluorethane fibers	Pb	213	0.26	1.0M HNO3	49	FAAS	environmental and biological samples	^49^
Cu(II)-imprinted styrene–divinylbenzene	Cu	9.55	1.03	0.5% HNO3	12	FAAS	Water sample	^50^
Silica gel	Pb	27.1	0.60	20.0 mL of 0.10 mol/L HNO3	—	FAAS	Water sample	^51^
Multi-walled carbon nanotubes	Pb	—	0.0028	0.3M HNO3	26	HG-AFS	water sample	^52^
GO–silica	Cu, Pb	—	0.023, 0.028	0.1M (0.5 M HNO3)	120	ICP-MS	Environmental water sample	^53^
PS GO	Pb	227.92	2.3	5 mL (2M HCl)	400	FAAS	Water and food	^23^

^a^sorption capacity, ^b^limit detection, ^c^preconcentration factor.

## Method Validation

The validity of a new method is checked by assessing parameters such as calibration, precision, limit of detection (LOD), limit of quantification (LOQ), accuracy and robustness under optimized column conditions. The linearity of calibration curve plotted by least square method after preconcentrating the series of standards for Pb (II) and Cu (II), was reported linear having correlation coefficient (R^2^), regression equation; and concentration range (µgL^−1^) as 0.9953, A = 13.9212*x_pb_ + −0.0567, 6.7–20000 and 0.9952, A = 1091.4904*x_cu_ + 2.0125 and 6.7–5000 respectively. The relative standard deviation characterizing for precision of the proposed method, analyzing for five replicate synthetic samples of 5 µg Pb (II) and Cu (II) in 50 mL, were found to be <5%. IUPAC defined (LOD) and (LOQ) as 3 S/m and 10 S/m^39^ of mean blank signal for 20 replicates, were found to be 1.434, 4.782 µg L^−1^ and 0.048, 0.160 µg L^−1^ for Pb (II) and Cu (II) respectively. Accuracy, assessed by analyzing an SRM following Student’s t test values calculated for Pb (II) and Cu (II) were less than the critical Student’s t value of 4.303 at 95% confidence level for N = 3 (Table 3). The observed mean concentration was not significant statistically when compared with the certified values, describing the absence of systematic errors. Reliability is strongly requested for the developed method which was studied by spiking with a known amount of 5 µg of analyte into the different fish samples and battery waste water. The percentage recoveries were checked for the spiked analytes and found to be (95–103.82%) having a relative standard deviation (RSD) < 5% in maximum results (Table 4).

**Table 3:** SRM analysis under optimized column conditions.

SRM	Certified value (µg g^−1^)	Found value (µg g^−1^)^a^ (RSD)	Calculated Student’s t test value^b^
Rompin hematite JSS (800–3)	Pb: 210	Pb: 203.8(1.63)	Pb: 3.21
	Cu: 640	Cu: 621.8(1.37)	Cu: 3.69

^a^Mean value, ^b^95% confidence limit, N = 3.

**Table 4:** ICP-OES determination of Pb (II) and Cu (II) in fish tissues samples and battery effluent after Column Preconcentration under optimized conditions.

Samples	Amount spiked Pb(II), Cu (II) (µg)	Amount found Pb (II) (µg g^−1^)	Mean %recovery Pb (II) (µg g^−1^)	RSD^a^ N = 3	Amount found Cu (II) (µg g^−1^)	Mean %recovery Cu (II) (µg g^−1^)	RSD^a^ N = 3
***Labeo rohita***							
Kidney	0	0.85	—	4.55	0.21	—	4.02
	5	5.47	95.6	2.4	5.3	98.6	2.73
	10	10.54	104.4	2.59	9.88	96.8	1.81
Liver	0	0.18	—	4.5	0.27	—	3.46
	5	5.22	102	1.31	5.14	95	4.4
	10	10.68	98.3	4.53	10.47	101.6	3.31
Muscle	0	0.14	—	4.44	0.12	—	4.09
	5	5.1	95.6	3.99	5.07	97.7	1.07
	10	9.64	100.2	2.69	9.49	98.4	3.98
Brain	0	0.65	—	4.95	0.22	—	4.14
	5	5.58	98	2.45	5.27	100.1	2.45
	10	10.96	100	3.43	10.7	103.5	4.91
Gills	0	0.21	—	3.31	0.12	—	3.28
	5	5.11	95.9	3.64	5.18	97.3	3.62
	10	9.93	104.8	3.5	10.01	100.9	2.06
***Channa punctatus***							
Kidney	0	8.66	—	5.21	0.47	—	5.3
	5	13.61	102	3.88	5.4	98.8	1.29
	10	17.93	98	2.84	10.42	104.1	2.74
Liver	0	6.34	—	4.21	1.35	—	4.94
	5	11.16	97.8	1.87	6.34	96.9	3.88
	10	16.67	96.7	4.61	11.67	95.1	4.85
Muscle	0	1.09	—	5.5	2.35	—	5.4
	5	6.06	98.74	2.05	7.32	103.8	4.26
	10	11.25	102.9	1.56	13.22	104.6	3.73
Brain	0	9.8	—	3.9	0.69	—	4.91
	5	15.01	102.4	4.44	5.73	98.8	3.45
	10	20.24	101.2	3.35	10.13	95.6	2.28
Gills	0	13.47	—	4.3	0.56	—	1.32
	5	18.37	99.54	2.14	5.14	96.7	4.19
	10	22.81	96.4	2.99	10.58	98.1	2.94
**Battery waste**							
Effluent	0	1.76	—	4.13	1.1	—	4.54
	5	6.85	95.8	2.86	6.25	101.8	1.97
	10	12.23	103.3	3.71	11.45	99.7	3.5

Robustness of the method was checked by tuning the pH 5.6 to pH 5.6 ± 0.5 and flow rate 5 to 5 ± 0.5 mL min^−1^ of the solution no significant change in the recovery was found (>96%) for Pb (II) and Cu (II) respectively.

## Materials and Methods

### Reagents and solutions

All chemicals were procured from the analytical reagent. Graphite powder (50 µm) was obtained from Otto Chemie Pvt. Ltd. (Mumbai, India). Amberlite XAD-16 (AXAD-16) (particle size 20–60, pore size 100 Å, pore volume 1.82 mL g^−1^, and surface area 800 m^2^ g^−1^) and (2-picolylamine) were purchased from Sigma-Aldrich (Steinem, Germany). All aqueous solutions were prepared in deionized water. Rompin hematite JSS (800-3) obtained from the Iron and Steel Institute of Japan (Tokyo, Japan) was used as a standard reference material (SRM). Buffer solutions were prepared by suitable amounts of HCl-glycine for pH 2.6, acetic acid-sodium acetate for pH 3.6–5.6 and ammonia-ammonium chloride for pH 8.0–10.0. Metal stock solutions of Pb (II) and Cu (II) (1000 mg L^−1^) were purchased from Merck (Darmstadt, Germany). HNO_3_, HCl, HClO_4_ and H_2_O_2_ were obtained from Merck (Mumbai, India).

### Instrumentation

#### Optimized column procedure for preconcentration/determination of metal ions

A column was packed with 0.1 g of GOXPA resin. The column was preatreated by passing 5 mL of buffer solution at pH 5.6 before subjected to experimental studies. Sample solution containing 5 µg Pb (II) and Cu (II) having pH 5.6 was allowed to pass through the column at a flow rate of 5 mL min^−1^. Elution of sorbed metal ions were done by 5.0 mL of 2M HCl followed by determination with ICP-OES. For the determination of Pb (II) and Cu (II) an inductively coupled plasma optical emission spectrometer (ICP–OES), Thermo Scientific (iCAP 7000 series, Waltham, USA) was used at the wavelengths 220, 353 and 324.754 nm respectively. Glass column 15 mm (8 mm × 100 mm) used for experimental studies obtained from J-SIL Scientific Industries (Agra, UP, India). The pH meter from Thermo Scientific (Orion 2 star, Waltham, MA, USA) was used for pH measurement. FT-IR spectral studies were analysed on a PerkinElmer Spectrum (Waltham, MA, USA), using a KBr disk method lies in the range between 500 and 4000 cm^−1^ with the resolution of 2.0 cm^−1^. Quantachrome Instrument (Boynton Beach, FL, USA) for Brunauer-Emmett-Teller (BET) surface area analysis. Shimadzu TGA/DTA simultaneous measuring instrument, (DTG-60/60 H, Kyoto, Japan) was run for thermo gravimetric analysis (TGA) and differential thermal analysis (DTA) at temperatures of 50−600 °C at a 10 °C min^−1^ heating rate and kept at an inert atmosphere (N_2_ flow rate of 50 mL min^−1^). Scanning electron microscopy (SEM) and energy dispersive X-ray analysis (EDS) (Jeol JSM-6510LV, Tokyo, Japan) was used after Coating with gold over layer to perform microstructural observations of the surface morphology and compositional analysis of the resin. Transmission electron microscope (TEM) images were obtained by using a Jeol JEM-2100 microscope (Peabody, MA). ^13^C NMR was recorded by Bruker BioSpin GmbH (Rheinstetten, Germany).

### Analysis of real samples and SRM

#### Ethical statement

Experiments on fishes for research purpose identifying the licensing committee approving the experiments, registration no. 714/02/a/CPCSEA issued and approved by the Institutional Animal Ethic Committee (IAEC) with Order no: D. No. 4165, by the Department of Biochemistry, Faculty of Life Sciences, Aligarh Muslim University, Aligarh, India. It has also been confirmed that all experiments were performed in accordance with relevant guidelines and regulations.

Battery waste water (Aligarh, India) was collected and filtered through cellulose membrane (Millipore) having a pore size of 0.45μm, acidified to pH 2.0 with HNO_3_ acid and kept in a precleaned polyethylene bottles. Samples of fishes *Labeo rohita* (n = 10, length 14.8 ± 2 cm, total weight 2 kg) and *Channa punctatus* (n = 5, length 13.4 ± 2 cm, total weight 1 kg) were collected from Panethi reservoir (Aligarh, India). Fishes were dissected and different organ tissues (muscle, kidney, liver, brain, gills) were separated and digested as reported in the literature^40^. The digestion of an SRM (JSS 800-3), rompin hematite was also reported in the literature^38^.

### Immobilization of GO onto solid support AXAD-16

Graphene Oxide (GO) was synthesized from natural graphite powder by modified Hummers method^41^. The diazotization of AXAD-16 resin was reported in the previous literature^40^.

### Synthesis of GO AXAD-16

Subsequently, GO was chemically immobilized onto surface of AXAD-16 resin through azo coupling. Briefly, 5 mL of prepared GO (2 mg mL^−1^) and 4 g of diazotized resin were added into the beaker containing methanol and NaOH (2:1) and then the mixture was kept at 0–2 °C for 48 h during this time GO was reacting with diazotized resin. Afterwards, the products were filtered and washed with deionized water several times. Finally, the (GO AXAD-16) were dried under vacuum at 50 °C for 24 h.

### Functionalization of (GO AXAD-16) with picolylamine

**Figure 5:**
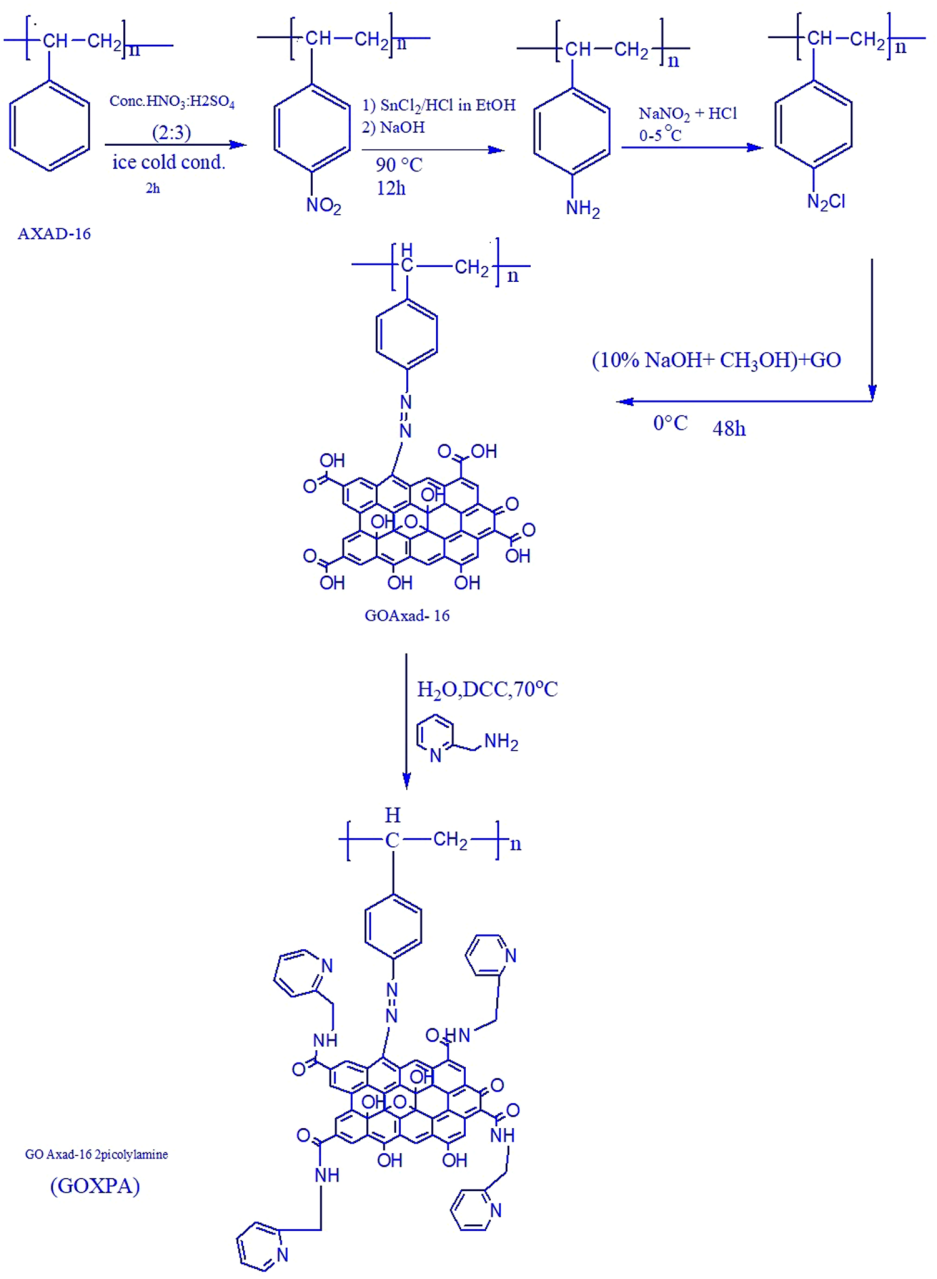
Representing immobilization of GO onto AXAD-16 resin through azo spacer arm and coupling via carboxyl group of GO and –NH_2_ group of ligand picolylamine.

In a 250 mL round bottomed flask, the synthesized GO AXAD-16 resin was dispersed into 50 mL DMF solution. Then DCC (1 g) and 2 picolylamine (2.5 mL in 30 mL aqueous solution) were added into the above mixture under heating at 70 °C for 8 h. Finally, the mixture was filtered and washed with deionized water with several times until pH become neutral. Lastly, the product was dried. The final product was abbreviated as GOXPA. As represented in the scheme (Fig. 5).

## Conclusion

The objective of the work was to engineer a novel and innovative solid phase extractant GOXPA in order to develop an environmentally safe column method for preconcentration and determination of Pb (II) and Cu (II) coupled with ICP-OES. As this involved immobilization of GO onto the surface of AXAD-16 resin through azo spacer arm without utilizing any oxygen functionality, which was revealed by sharp azo peak in FT-IR spectrum and through SEM images of disordered covering of GO onto AXAD-16 resin and further scrolling of GO was supported by TEM image. ^13^C NMR of GOXPA was also analyzed and observed a chemical shift in the spectrum, confirming successful immobilization and modification. The higher surface area was achieved by utilizing AXAD-16 resin as a solid support which was investigated by BET analysis. Hence, this engineered sorbent has efficacy for preconcentration and determination of Pb (II) and Cu (II). Moreover, it was successfully applied to both different tissues of animals like fishes as well as water sample which confirmed that the method was reliable with good accuracy and precision.

## Electronic supplementary material

Supplementary information

## Data Availability

All the described data is available in the manuscript.
