# Supplementary material for: Efficacy of engineered GO Amberlite XAD-16 picolylamine sorbent for the trace determination of Pb (II) and Cu (II) in fishes by solid phase extraction column coupled with inductively coupled plasma optical emission spectrometry
Source: Sci Rep. 2018 Dec 3;8:17560. doi: 10.1038/s41598-018-35656-1 (PMC6277385; doi:10.1038/s41598-018-35656-1)
Supplement: Supplementary file 1 — Supplementary information [file 41598_2018_35656_MOESM1_ESM.docx]

**Supplementary information**

**Efficacy of engineered GO Amberlite XAD-16 picolylamine sorbent for the trace determination of Pb (II) and Cu (II) in fishes by solid phase extraction column coupled with inductively coupled plasma optical emission spectrometry**

**Hina Javed^a^, Aminul Islam^a*^, Anjali Chauhan^a^, Suneel Kumar^a^, Sushil Kumar^b^,**

^a^Analytical Research Laboratory, Department of Chemistry, Aligarh Muslim University, Aligarh, India 202002

^b^School of Environmental Sciences, Jawaharlal Nehru University, New Delhi, India,
110067

*Correspondence to: [aminulislam.ch@amu.ac.in](mailto:aminulislam.ch@amu.ac.in)

**
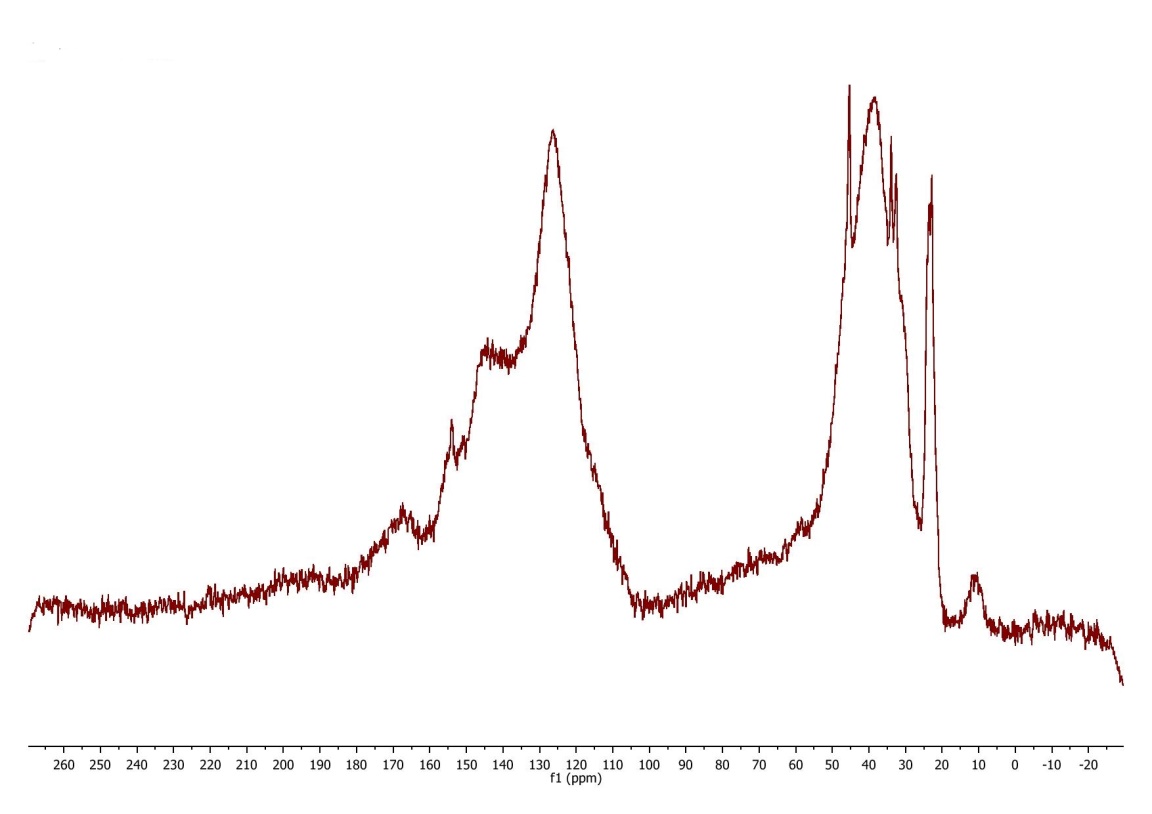
**

**Figure S1** ^13^C NMR spectrum of GOXPA


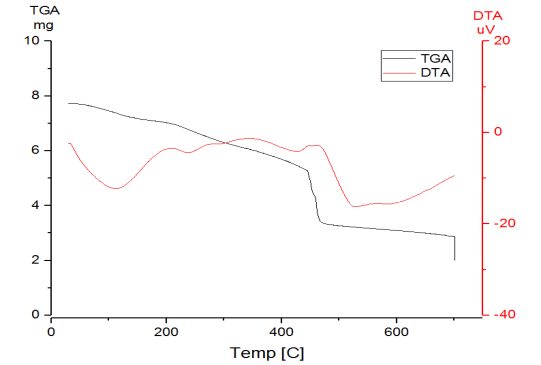


**Figure S2** TGA DTA spectrum of GOXPA

**Figure S3** showing effect of pH on sorption of Pb (II) and Cu (II)
